# The CBC theory and its entailments: Why current models of the origin of consciousness fail

**DOI:** 10.1038/s44319-023-00004-6

**Published:** 2023-12-13

**Authors:** Arthur S Reber, William B Miller, Predrag Slijepcevic, František Baluška

**Affiliations:** 1 Department of Psychology, University of British Columbia, Vancouver, BC Canada; 2 Bioverse Foundation, Paradise Valley, AZ USA; 3 Department of Life Sciences, College of Health, Medicine and Life Sciences, University of Brunel, Uxbridge, UK; 4 Institute of Cellular and Molecular Botany, University of Bonn, Bonn, Germany

**Keywords:** Evolution & Ecology, History & Philosophy of Science

## Abstract

The Cellular Basis of Consciousness (CBC) model of biological consciousness is based on the assumption that life and conscious sentience are coterminus. All living organisms, are conscious, self-aware, and have valenced sensory and perceptual experiences.

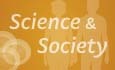

Consciousness represents one of the most mysterious aspects of life. There are about 50 diverse theories behind consciousness and one of the two currently oft-accepted theories, known as the integrated information theory (IIT), was recently declared by a group of 124 scholars, including Bernard Baars, Daniel Dennett, and Joseph LeDoux, to be a pseudoscience with a panpsychic slant that is having damaging effects on biology (Fleming et al, 2023; Lenharo, 2023). This accusation caused a considerable uproar and prompted other experts on consciousness, including Christoph Koch and Anil Seth, to defend the IIT concept (Lenharo, 2023).

IIT proposes a mathematical model to explain why some systems, such as brains, are conscious. Generally, it assumes that the model could infer from objective and causal properties whether a given system is conscious, to what degree and what particular experience it is having. We have discussed IIT elsewhere (Reber, 2019; Reber et al, 2023) and will briefly note here that there are serious theoretical weaknesses. Most stem from the assumption that the various parts of a system must be *integrated* for it to become sentient. As Reber (2019) pointed out, this component of the model ends up producing nonsensical situations where a pile of sand or a camera would become sentient under the right circumstances. For example, if the pile of sand is in a beaker and it is shaken, the grains, being of slightly different diameters would undergo a vertical sorting. In short, they would now be interacting with each other, but we would not conclude that there is an existential consciousness in the beaker. An insentient camera should, according to IIT, become conscious when it is integrated into a moving vehicle that is equipped with the sensors and feedback systems found in self-driving cars. There are other problems with the overall theory, including a lack of any coherent way to measure the amount of “information” that is in any system or determine precisely what it means for it to be “integrated”.

In order to keep consciousness a legitimate research field, we need a new fresh look on the mystery of consciousness, which our Cellular Basis of Consciousness (CBC) theory offers without becoming enmeshed in the kinds of conundrums that the IIT invites. CBC differs in important ways from that taken by others in the broad field of consciousness science—a stance we’ve labeled the Standard Model of Consciousness (SMC).

## The CBC framework

The framework for the Cellular Basis of Consciousness (CBC) model was first introduced in the 1990s (Reber, 2019). The CBC theory is based on the assumption that life and sentience are coterminous. All, but only, living organisms are conscious, self-aware, and have valenced sensory and perceptual experiences (Baluška and Reber, 2019). Prokaryotes, the simplest unicellular species, display behaviors that are clearly cognitive in nature including associative learning, stable memory formation, route navigation and decision-making. They anticipate upcoming events and readily create functional social collectives, within which they display both cooperation and competition and, fascinatingly, a primitive form of altruism where some cells in a colony put themselves at risk to support the life functions of other cells in distress (Reber et al, 2023).

Prokaryotes, the simplest unicellular species, display behaviors that are clearly cognitive in nature including associative learning, stable memory formation, route navigation and decision-making

Interestingly, estimates of the initial emergence of life are based on fossils of microbial mats, calcareous accretions produced by the metabolic activities of communities of ancient prokaryotes. The earliest single-cell organisms left no fossils; only these mats were large enough to leave evidence of their existence. Estimates based on the geological record put this event as having occurred at least 3.4 billion years ago. It is important to recognize that assembling and surviving as functioning collectives require directed, communal action as a form of cellular problem-solving (Miller et al, 2023).

As discussed in Slijepcevic (2023), this cellular communal action provided the basis for various forms of social intelligence. The term *biocivilizations* was recently coined to recognize this continuity of social intelligence across all kingdoms of life which manifests in social skills such as communication, engineering, and agriculture (Slijepcevic, 2023). All have roots in the cognitive capacities of the first cells—bacteria and archaea. Necessarily then, the first live coincided with cognitive competence.

It is simply inconceivable that this wide range of cognitive functions could be the result of a cluster of “dumb” gene-driven mechanisms. But many who embrace the SMC maintain that this is, in fact, the case. Philosopher Daniel Dennett (2017) referred to them as displays of “competence without comprehension”. However, Dennett never makes an effort to identify the mechanism(s) through which such skills could have evolved and how they became to be instantiated in ancient prokaryotes. It is improbable as each of these several “competencies” would have had to emerge through genetic manipulations independent of each other—one for learning, another for sensory mechanisms, yet another for perceptual processes, one for building functional colonies, and others for each of the abilities noted. On the other hand, recognizing that the very first, simple, unicellular species were sentient, self-aware and capable of decision-making —in short endowed with the kinds of cellular intelligence and cognition displayed in the form of Dennett’s “comprehension”—and the evolutionary picture becomes clear. All life is sentient life.

## The SMC framework

The CBC theory is not the only and not the generally accepted framework within which consciousness science operates. The approach taken by the majority of scientists and philosophers in the field begins with the mental life of *Homo sapiens* and follows two traditional research strategies. In the SMC framework, the focus is on identifying behavioral characteristics that are considered diagnostic of human cognitive functioning. Once there is general agreement on what these are, a search through the evolutionary tree is carried out with an eye to other species that can also be seen as having these functions or at least clear precursors of them. The other research path focuses on identifying the underlying neurological pathways and centers in the human brain that are responsible for these functions. As with the behavioral strategy, the search is on for other species that have equivalent neurological structures or homologs or analogs of these.

These research programs have made intriguing and important discoveries about the cognitive functions of a host of species which, only a few decades ago, were deemed to be insentient. But there has been virtually no progress in the search for the origins of consciousness. The reason is simple: the SMC model is not an optimal strategy because it starts at the evolutionary end, with the most cognitively sophisticated species, not at the beginning of evolution where sentience first emerged.

… the SMC model is not an optimal strategy because it starts at the evolutionary end, with the most cognitively sophisticated species, not at the beginning of evolution where sentience first emerged.

## Terminology

A good deal of the confusion that exists in the field can be traced to lexicographic issues. An example came from a recent publication by neuroscientist Antonio Damasio (2023) who, while acknowledging the remarkable range of behaviors that have been observed in prokaryotes, maintained that they weren’t expressions of a “true consciousness”. He is not alone in taking this stance − Dennett’s distinction between competence and comprehension discussed above is similar in nature. It’s easy to see the problem: it is not about the science, it is about the labels being used when talking about the science.

This terminological tangle results from the adoption of the SMC by most researchers in the field. Starting with human minds invokes a bias that awards our human form of consciousness a special, distinct, and superior status; one that is different in fundamental ways from the mental experiences of other species. Other species may—often grudgingly—be determined to have an existential consciousness but it takes a solid empirical database to persuade colleagues. Amusingly, as we have pointed out elsewhere (Reber et al, 2023), the assignment of a mind to a species or clade—accompanied by the use of terms like “sentience” and “cognition”—is typically made by the research team that has done the empirical work. Entomologists have presented compelling evidence that many insects are conscious and self-aware; avian specialists are comfortable using “consciousness” when referring to the behaviors displayed by many bird species, especially corvids; those who study cephalopods have determined that octopuses and cuttlefish have palpable minds. The reason, of course, is that consciousness has been there from the very beginning of life. All they had to do was look.

Other species may—often grudgingly—be determined to have an existential consciousness but it takes a solid empirical database to persuade colleagues.

In our work, we have taken a “folk psychology” approach to these terminological issues. Because there is such a wide variety of ways in which these terms are used in the field, we treat them all as loose synonyms of each other. In both *The First Minds* (Reber, 2019) and *The Sentient Cell* (Reber et al, 2023), we have appendices outlining the various lexicographic issues that have arisen over the years and explain why using them loosely is an advantage and avoid that common retort in philosophy: “Excuse me, could you please define your terms, what exactly do you mean by ____.” (Fill in the blank as you wish.)

## The first-person problem

A reasonable question to ask is how and why the field adopted the SMC in the first place. The issue emerged with what’s known in philosophy as the “First-Person Problem”. In its simplest instantiation, it simply points out that the only entity that one can be absolutely certain has consciousness is oneself. An individual cannot know what is in anyone else’s head. But we can be generous. If others look like ourself, and do many of the same things, it is not unreasonable to grant others a mental life that is similar to ours. In recent years, consciousness has been granted to several species of animals, especially to mammals and birds.

Once this step is taken, the SMC becomes the only way to go about exploring the evolutionary framework looking for the place where this consciousness thing runs out, where we encounter the species for which we can no longer be generous, the one where the behavioral repertoire and/or the underlying neurological apparatus no longer seems close enough to ours to conclude that it is sentient. But, as we just pointed out, this approach fails. It must, as there are no species without consciousness.

## Why neurons—why a nervous system?

Many who work within the SMC assume that a nervous system is necessary and sufficient for an existential consciousness. While this is a common stance—it underlies Damasio’s arguments noted above—we have yet to see a coherent defense of this proposition or a well-developed biomolecular argument for it. For most, it is simply a proclamation. Moreover, we have not seen any effort to identify what features of neural mechanisms “create” consciousness while non-neural ones cannot. This too is simply a pronouncement.

We appreciate that when neural structures emerged, when neural pathways and centers formed, when brains evolved, there were many changes in functions but the evolutionary origin of consciousness, of cognitive functioning was surely not one of them. Many evolutionary changes produced dramatic gains in functioning. Photosynthesis, extraction of oxygen from water and later air, spectral vision, rapid locomotion, first in water, later on land, flight … the list is long. But these all had precursors in ancient bacteria and archaea. The reason no one has identified the biomolecular mechanisms that allow neural systems to cause sentience to emerge in previously insentient species is simple. It did not happen.

## The emergentist’s dilemma

Lurking behind many of these problems with the SMC is what we have dubbed the “Emergentist’s Dilemma”. Put simply, if avian species were the first to be sentient how did this nearly miraculous event take place when the species from which they evolved were dumb as rocks? What were the biological mechanisms of neurons that allowed consciousness to piggyback on them?

As with the previous conundrums that accompany the SMC stance, we have yet to see this issue confronted. Appreciate that when the explorations begin with human consciousness you’re not really exploring the great expanse of species looking for the point where consciousness emerged—you are actually looking for the point in the evolutionary tree where it “runs out”, where it can no longer be found. Once this becomes the goal, the issue of what biomolecular elements make sentience, self-awareness, valenced perceptions possible lie outside the SMC conceptual framework (Reber et al, 2023).

We acknowledge that, in assuming the CBC stance, we have our own emergentist’s dilemma. In our recent work (Baluška and Reber, 2019; Miller et al, 2023; Reber et al, 2023), we have examined a variety of biomolecular functions and mechanisms in an effort to identify those that are responsible for creating sentience in cells. Briefly, they are almost certainly *holistic* in the sense that there are various tightly linked functions involving the cytoskeleton, the cell membrane including specific sensors and receptors, and the mechanisms that permit molecular exchanges between the interior of the cell and the external environment. All of these and others combine to allow the cell to detect, evaluate and mentally represent events and objects and make appropriate decisions about how to respond to them. How these biomolecular processes evolved to create the first living, sentient species is a topic of considerable research, much of it carried out under the “origins of life” research banner. The search is for, in Israeli biochemist Addy Pross’s trenchant phrase, the point where “chemistry becomes biology” (Pross, 2012).

How these biomolecular processes evolved to create the first living, sentient species is a topic of considerable research, much of it carried out under the “origins of life” research banner.

## Plants, flora

One of the foundational principles of evolutionary biology is “if it works, it stays.” Traits that are adaptive and functional are rarely jettisoned. Rather, they become the platforms from which other, novel traits and forms evolve. One of the entailments of the CBC is that plants, which are eukaryotes and emerged late in evolutionary terms, are conscious, sentient beings.

The data are compelling. As outlined previously (Brenner et al, 2006; Trewavas and Baluška, 2011; Baluška et al, 2016; Baluška and Reber, 2019), there is considerable evidence for valenced sensation, decision-making, learning, and communication in flora. There is a vigorous sub-field within plant sciences dubbed *plant neurobiology* (Brenner et al, 2006) that explores the underlying mechanisms that support plant behavior, a peer-reviewed journal (*Plant Signaling and Behavior*), and an international society that holds regular symposia for scientists to inform and become informed of the latest research.

One of the primary reasons for the reluctance of those who work within the SMC framework to include flora is their conviction that a nervous system is a requirement for a genuine consciousness. However, much of the research into plant cognition supports the conclusion that plant root systems function in ways that are analogous of neural systems (Brenner et al, 2006; Baluška et al, 2016). Moreover, all plants are sensitive to anesthetics and many anesthetics used in medicine are derived from plants (Baluška et al, 2016). Why, one might ask, would a species waste precious natural resources to support the systems that detect and react to anesthetics if it didn’t have valenced experiences? If a species doesn’t feel pain why be sensitive to compounds that block the experience of pain? Why would plants generate their own endogenous anesthetics when heavily stressed or wounded if they were not sentient?

## Post neo-Darwinism

There is a growing sense of unease among biologists that there are serious shortcomings in the Neo-Darwinian framework, in particular that several of its central assumptions are wrong and that, as a result, it lacks explanatory power. The problems are many and likely fatal. For one, epigenetic effects are not only real, they are critical for the evolution of cells. Epigenesis had been largely excluded from the Darwinian paradigm due to Lamarckian theory having been deemed in error. Moreover, it is becoming increasingly clear that the central assumption of Neo-Darwinism, that mutations occur randomly and that natural selection operates to fix the most adaptive variations, is simply wrong (Miller et al, 2023). It is virtually certain that cells change the manner of gene expression by the decisions and choices they make (Shapiro, 2011). These epigenetic modifications are the driving force behind collaborative cellular problem-solving involved in dealing with environmental stresses.

We anticipate a shift from a gene-centric Neo-Darwinism and SMC to a cognition-centric CBC framework (Miller et al, 2023). The result will be an evolutionary biology based on systematic, natural learning carried out by intelligent and sentient cells—not on random genetic errors. Importantly, all multicellular organisms evolved from ancestral unicellular organisms (Baluška et al, 2022). As noted above, these cells still retain their organismal, semi-autonomous nature and use their cellular intelligence and sentience in the development and evolution of all species of fungi, plants, and animals—including us humans. Neo-Darwinists, working within the SMC, typically miss this critical point because they tend to ignore unicellular organisms, including archaea, bacteria, and protists. We are the terminal points of some 4 billion of years long continuous evolutionary cellular history.

## The CBC framework bridges the intense disagreements

In formulating ITT, Giulio Tononi introduced a theoretical measure of the potential integration of information, designated by the Greek letter phi (Ф), which expressed the degree to which the system’s separate components interrelate. In this frame, consciousness depends on the “informational relationships generated by a complex of elements”, that is, a sufficiently high Ф (Tononi, 2004). Thus, consciousness is not just a matter of the amount of information but explicitly relates to its interconnectedness, and this ‘integration’ can be considered a measure of consciousness. However, without placing sufficient constraints within that theory, any sufficiently complex non-living system might be deemed conscious.

These are very serious issues, to be sure. However, despite the asperity of the critical comments in the open letter mentioned in our introduction, it should be noted that none of the competing alternative theories provide definitive answers to the enigma of consciousness. The problem remains intractable since consciousness is privately self-referential by definition and thereby unsuited to outside measurement. Noting this issue, Goff (2020) contests that ITT does not explain consciousness, but even so, that does not equate to assuming it is without value. What is needed to make progress on the mystery of consciousness is an openness to innovative thinking.

The problem remains intractable since consciousness is privately self-referential by definition and thereby unsuited to outside measurement.

In that regard, our CBC model stands apart from current contending theories but recognizes that each has its potential merits. Pertinently, the CBC framework overcomes several specific problems of the ITT framework by placing discrete constraints on some of its insights and clarifying how information is integrated in the living frame. ITT in fact offers five specific elements that are applicable to CBC (Oizumi et al, 2014). First, it asserts that consciousness exists and that consciousness is structured, and that every experience is a combination of its aspects. Consciousness is informative, permitting the discrimination of experiences. Consciousness is integrated to such an extent that each experience cannot be reduced to its components to be understood. Finally, each experience is exclusive and unlike any others.

As we have noted, the origin of consciousness and life were co-terminus, becoming epitomized within the cellular form. Correspondingly, all cells are sentient, exhibit self-referential awareness, and are fully capable of decision-making and problem-solving. These cellular faculties rely on whole-cell integrated information, including its reception and its internal assessment enabling the self-referential decision-making that determines the contingent expenditure of limited cellular resources (Baluška et al, 2022).

Accordingly, each cell is a conscious “self”, combining three essential elements necessary for cogently explaining multicellularity. In order to collaborate in their trillions, each self-referential cell must “know that it knows”, “knows that others know”, and be aware that other cells “know in self-similar patterns”. These aspects of consciousness are essential to the collaboration, cooperation, and co-dependencies that cells demonstrate for multicellular decision-making and united contingent problem-solving.

Within CBC, consciousness exists because it is instantiated in the cell in a replete basal form. Each of the five elements that ITT suggests are required for consciousness that directly pertains since all cells self-produce their own information (Miller et al, 2023). All environmental stimuli must be assessed within cells. Consequently, cellular information is exclusive by definition since it is self-generated through internal measurement. Thus, cellular information is valenced, self-referential, and separated according to each individual cell’s connection to information space-time (Miller et al, 2023). However, CBC clarifies that whereas the five elements may constitute *necessary* descriptive features of the attributes of consciousness, they are not themselves *sufficient*.

Crucially, CBC imposes three essential requirements to specifically enable cell-wide integrated information as consciousness. Cellular consciousness requires boundaries (a plasma membrane), a cell-wide integrative apparatus for the reception and internal assessment of environmental information (its senome) and linked retrievable and deployable memory (Baluška and Miller, 2018, Miller et al, 2023; Reber et al, 2023). Effective integrated information can only occur when those features are present. Thus, although information may be universal, consciousness is local. Only living cells meet these requirements for consciousness. Physical systems do not.

Within CBC, consciousness becomes the channeled aggregation of that local consciousness to become the varieties of multicellular consciousness (Reber, 2019). Consequently, it would be expected that that same system-wide integration of information that exists within individual cells would manifest within multicellularity.

Pertinently, experiments confirm that correlation. The spatiotemporal distribution of sensory inputs such as sight, thinking, or dreaming does not localize within any single brain location. Instead, there is widespread information integration across our brains, suggesting that consciousness is a diffuse phenomenon as advanced within the Global Neuronal Workspace Hypothesis (Mashour et al, 2020).

We argue that CBC is the natural bridge among all competing theories. Minds do not create conscious self-awareness (Reber, 2019; Miller et al, 2023). Minds, such as our own, are an aggregation of individual cellular consciousnesses. Thus, any theory of the human mind must begin with a deep exploration of cellular consciousness, from the bottom forward, rather than the reverse, which heretofore has been the dominant direction. That search must first begin with a deeper understanding of the basic cell and then further delve into their means of uniting into a seamless ensemble to become our unique human form of conscious problem-solving.

## Coda: we are not alone—similar views

Finally, it is important to keep in mind a foundational principle of evolutionary biology with which there is virtually universal agreement: life emerged once on planet Earth and all species, extinct and living, evolved from these original unicellular *ur*-species. What the CBC stance maintains is that sentience, cognition, and consciousness (whatever term you wish) followed the same evolutionary path. Those ancient prokaryotes were sentient and all the species that evolved from their original biomolecular platform were and are sentient. We anticipate that this perspective will continue to attract attention within the field and that, as more researchers pursue the entailments of the CBC, insights and understanding will follow. We look forward to a paradigm shift in evolutionary biology.

Those ancient prokaryotes were sentient and all the species that evolved from their original biomolecular platform were and are sentient.

### Supplementary information

Peer Review File
