# Supplementary material for: The CBC theory and its entailments: Why current models of the origin of consciousness fail
Source: EMBO Rep. 2023 Dec 13;25(1):8–12. doi: 10.1038/s44319-023-00004-6 (PMC10883262; doi:10.1038/s44319-023-00004-6)
Supplement: Supplementary file 1 — Peer Review File [file 44319_2023_4_MOESM1_ESM.pdf]

EMBO reports

The CBC Theory and it's Entailments: Why Current Models of the Origin of Consciousness all Fail

Frantisek Baluska, Arthur Reber, William Miller Jr., and Predrag Slijepcevic
DOI: 10.15252/embr.202358211

Corresponding author(s): Frantisek Baluska (unb15e@uni-bonn.de) , Arthur Reber (areber@pointroberts.net)

Review Timeline:

Submission Date:	25th Sep 23
Accepted:	9th Nov 23

Editor: Holger Breithaupt

Transaction Report: This article was editorially reviewed.
